# A quantitative cross‐sectional analysis of the melanin index in the skin of preterm newborns and its association with gestational age at birth

**DOI:** 10.1111/srt.12810

**Published:** 2019-11-25

**Authors:** Paola Conceição Silva, Rodney Nascimento Guimarães, Rayner Guilherme Souza, Zilma Silveira Nogueira Reis

**Affiliations:** ^1^ Faculty of Medicine Postgraduate Program in Women’s Health Universidade Federal de Minas Gerais Belo Horizonte Brazil; ^2^ Faculty of Medicine Center of Health Informatics Universidade Federal de Minas Gerais Belo Horizonte Brazil; ^3^ Faculty of Medicine Universidade Federal de Minas Gerais Belo Horizonte Brazil

**Keywords:** gestational age, infant, premature, photomedicine, physiological phenomena, pigmentation, skin

## Abstract

**Background:**

Estimation of gestational age (GA) is important to make timely decisions and provide appropriate neonatal care. Clinical maturity scales to estimate GA have used skin texture and color to assess maturity at birth facing situations of the uncertainty of pregnancy dating. The size and darkness of the areola around the nipple to grade skin characteristics are based on visual appearance. The melanin index (M‐Index) is an optical skin parameter related to the melanin content in the tissue. This study is aimed to associate the M‐Index of the skin with the GA.

**Methods:**

A cross‐sectional study evaluated 80 newborns at birth. A photometer device quantified the skin pigmentation on the areolae, forearms, and soles. Paired average differences of M‐Index were compared among the three body sites. The skin M‐Indexes were compared between subgroups of newborns until 34 weeks or with 34 and more.

**Results:**

The skin over the areola had the highest values of M‐Index compared with the forearm or sole areas (*P* < .001 for both). Infants with a GA between 34 and <37 weeks had higher M‐Index values over the areola than the group with a GA with 24 to <34 weeks: 41.7 (8.9) and 38.3 (10.5) median (IQR), *P* = .005.

**Conclusions:**

The measurable M‐Index values have the potential to improve physical evaluation in assessing GA at birth.

## 1 INTRODUCTION

Physical characteristics of neonates have been used to estimate maturity as they depend on the duration of pregnancy.^1^ Clinical score systems consider skin texture and color as signals to estimate gestational age (GA) after birth. Among a set of neurological testing and physical signs, health professional grade the size and darkness of the areola around the nipple based on visual appearance.^2^, ^3^, ^4^ However, under visual inspection to the color assessment of skin is subjective, sometimes clinically inadequate, and discordant between observers.^5^ In such evaluation, the color of the skin plays a major role despite being a subjective perception.

Skin color is the result of the existence of two main cutaneous chromophores, melanin and hemoglobin located in the epidermis and dermis, whose optical properties are well studied.^6^ Photometric methods to measure skin color evaluate the interaction of light with chromophores taking into account the optical properties of these compounds, their concentrations, and specific wavelengths of the illuminating source.^5^ The melanin index (M‐Index) is an optical skin parameter related to the melanin content in the tissue.^7^ In clinical practice, the phototype scale of Fitzpatrick et al is a semi‐quantitative measure of the skin color that combines the intensity of the melanization and the erythemal response to sun exposure.^8^

During the neonatal period, skin color is the result of pigments and skin thickness.^9^ Analyzing skin reflectance at birth, Post et al reported that, in utero life, black and white infants already show skin reflectance differences due to the sex and site of the body after 32 weeks of gestation.^9^ In fact, another report assessing the M‐Index in 447 healthy neonates from 35 to 42 weeks of gestation showed that the skin color of neonates is mainly determined genetically.^10^ Therefore, knowledge about physiological variations in skin pigmentation has clinical relevance because of associations with jaundice, anemia, plethora, and hormonal dysfunctions.^11^ Studies on the skin pigmentation of newborn infants are scarce or focused on the measurements of transcutaneous bilirubin,^12^ namely the influence of melanin in noninvasive measurements.^13^ Meanwhile, assessment of GA at birth supported by noninvasive photometry by skin contact is a new approach, which is under clinical validation. There is a potential association between skin reflectance and GA at birth.^14^ Post‐natal approaches to pregnancy dating have limitations regarding accuracy and precision.^15^ Preterm infants require special health care, mostly when they are born within <32 weeks of gestation, demanding inpatient care to survive. Innovations are still necessary to measure gestational age more efficiently in low‐income settings, considering low‐cost solutions.^16^

This study aimed to assess the M‐index of the skin in premature newborns and associate it with the GA at birth. We hypothesize that the darkness of the areola, assessed with a handheld photometer device, has the potential to contribute to pregnancy dating after birth in preterm neonates.

## 2 MATERIALS AND METHODS

### 2.1 Study design and setting

The design of the study was an observational cross‐sectional gathering of obstetric data, a clinical observation of the skin color in women, and an assessment of the M‐Index in the skin of newborns using a noninvasive optical device. Two referral perinatal centers in Brazil participated in this evaluation: Hospital das Clínicas, Universidade Federal de Minas Gerais, and Hospital Sofia Feldman. Each local independent ethics review board approved the study protocol. This study was logged in Plataforma Brasil under protocol number CAAE 49798915.2.0000.5149. Parents signed a written informed consent form on behalf of the newborns. Prospective data and skin signal collection occurred between August 2017 and September 2018.

A sequential enrollment process selected women and their infants with the following inclusion criteria: age newborn up to 24 hours of life with a GA between 24 and 36 weeks + 6 days at birth, confirmed with an obstetric ultrasound assessment before 14 weeks of pregnancy. Exclusion criteria were malformation with structural skin alterations or skin modifiers as follows: anhydramnios, hydrops, congenital skin diseases, or chorioamnionitis.

Inpatient medical records and a quick interview with the women were the sources of clinical data. The first‐trimester ultrasound was the reference for GA calculated at birth. Ranges of age: 24 to <34 weeks, 34 to <37 weeks bound two groups of analysis, according to the priorities of the newborn.^17^ The color of the mother's skin was measured from the inner forearm using the Fitzpatrick reference.^8^ This visual scale for skin phototypes ranged from 1 (pale light white) to 6 (very dark brown to black).

Neonates had their skin reflectance assessed at three body sites, the anterior distal forearm, the sole of the foot, and the areola. Choosing such areas, the examiner avoided any interference with vital signal sensors in use, ensuring minimum handling and stable clinical conditions in preterm infants, mainly inside incubators. The areola was assessed due to this importance as a clinical maturity marker.^1^, ^2^, ^4^

### 2.2 M‐Index measurement

**Figure 1:**
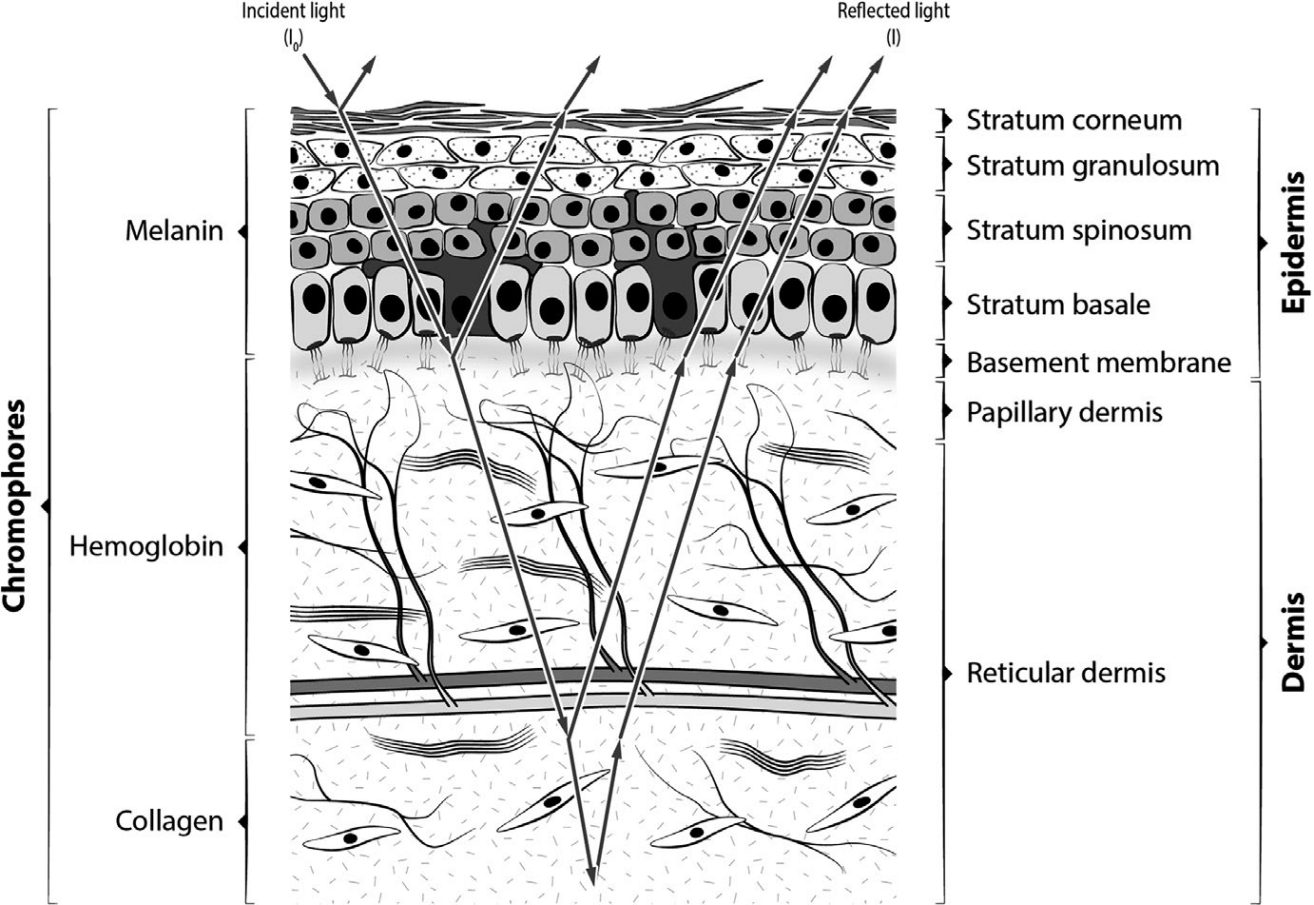
Simplified three‐layered optical skin model. Legend *I*, intensity of reflected light from a layered structure; *I*_0_, intensity of incident light

A handheld multiband reflectance photometric device, previously detailed,^18^ provided noninvasive acquisitions of skin reflectance under red LED light centered at 630nm. A simplified equation was used to relate skin reflection to the melanin concentration, according to Fullerton et al: M‐Index = 100 × Log_10_ (1/*I*_red_).^5^ A three‐layered optical skin model was adopted. The incident light (*I*_0_) enters the first layers of the skin and follows a tortuous path until it exits back out of the skin being absorbed by chromophores,^19^ see Figure 1. The intensity of reflected light (*I*) acquired by the photometer was then analyzed. The data acquisitions occurred automatically, when the sensor touched the skin, recording once per newborn and body site.

### 2.3 Data analysis

A descriptive analysis is expressed as the frequencies, the mean, and standard deviation (SD) presented baseline characteristics of the study group and skin M‐Index, whereas the median and interquartile range (IQR) were preferred for non‐normally distributed continuous variables. Independent mean or median tests, Student's *t*, Mann‐Whitney, or Kruskal‐Wallis, evaluate the relationship between the measurements and groups of interest. Paired *t* tests compared M‐Index differences among the three body sites of the infant. The significance level for hypothesis tests will be 5%, together with 95% confidence interval (CI). A sample size of 80 newborns had the power of 82% for detecting a mean difference between the two groups, considering a medium size effect (0.6) and 95% of confidence, in one‐tail t test.^20^

## 3 RESULTS

From among the 87 consented preterm newborns, 7 (8.0%) were excluded due to a technical problem of the sensor and producing invalid values. The median GA of 80 newborns was 34.3 (IQR = 3.7) and ranged from 24.1 to 36.9 weeks. We then stratified the infants by GA and found 30 (37.5%) were between 24 and <34 weeks, and 50 (62.5%) were between 34 and <37 weeks. Birth weight ranged from 510 to 2545 g; with a mean (SD) of 1967 (SD = 628) grams. Measurements were taken, 34 (42.5%) were female and 46 (57.5%) were male. The evaluation occurred inside an incubator in 36 (45.0%) infants and 37 (46.3%) infants were under intensive care. Sixteen newborns were siblings, and the number of mothers was 72 women. Most mothers had a phototype classification of type‐III (34/72 42.5%) or type‐IV (22/72 27.5%). Table 1 summarizes clinical characteristics of the groups stratified by GA.

**Table 1:** Clinical characteristics according to the groups of analysis

	24 to <34 wk n = 30	34 to <37 wk n = 50	*P*
Gestational age, weeks, median (IQR)	31.7 (2.8)	35.6 (1.9)	<.001†
Birth weight, grams, mean (SD)	1393 (424	2311 (453)	<.001‡
Color of the mothers skin¶, median (IQR)	4 (2)	3 (1)	.144†
Sex (female) n (%)	13 (43.3%)	21 (42.0%)	.907§
Incubator n (%)	28 (93.3%)	8 (16.0%)	<.001§

Abbreviations: IQR, interquartile range; SD, standard deviation.

^†^ Mann‐Whitney.

^‡^ Independent *t* test.

^§^ Chi‐square.

^¶^ Fitzpatrick scale

A total of 238 optical assessments on the skin were taken, 80 on the anterior distal forearm, 80 on the sole, and 78 on the areolar area. M‐Index mean (SD) values when evaluated on the anterior distal forearm were 33.5 (5.6); 35.4 (5.7) on the sole; and of 39.6 (7.6) on the areola. Two missing data were due to electrodes of ECG monitoring attached over areolae. The areola area was the darkest site of the three areas evaluated on the body of the newborn. Paired comparisons revealed significant within‐subject differences in the M‐Index on the areola compared with the forearm (Line 2 *P* < .001), and the sole (Line 2 *P* < .001), see Table 2.

**Table 2:** M‐Index taken from areola, forearm, and sole of preterm infants

Comparisons	Areola (n‐78)	Forearm (n = 80)	Sole (n = 80)	Mean‐paired difference (SD)	95% CI	*P* †
Areola minus forearm	39.6 (7.6)	33.5 (5.6)	—	6.3 (5.5)	5.0‐7.5	<.001
Areola minus sole	39.6 (7.6)	—	35.4 (5.7)	4.3 (6.4)	2.9‐5.8	<.001
Forearm minus sole	—	33.5 (5.6)	35.4 (5.7)	−1.9 (2.7)	−2.5‐1.3	<.001

Abbreviation: SD, standard deviation.

^†^ Paired *t* test.

When we stratified M‐Index values by sex of the neonate infant, no significant differences were found between the mean (SD) value on areola 38.9 (5.7) in females vs 40.2 (8.8) in males, *P* = .456; on forearm 34.2 (5.3) in females vs 33.0 (5.7) in males, *P* = .358; neither on the sole 35.8 (5.8) in females vs 35.0 (5.7) in males, *P* = .525.

Comparison of the skin color of the mothers had no statistical association with the color of the skin of the newborns assessed by M‐Indexes (Table 3). None of mothers had phototype classified as type‐I evaluated with the Fitzpatrick scale.

**Table 3:** Melanin index mean values measured at three sites of the skin of the newborns, according to the skin of the mother's phototype

Fitzpatrick scale	n (%)	Areola mean (SD)	Forearm mean (SD)	Sole mean (SD)
Type‐II	7 (8.8%)	34.3 (7.2)	29.5 (6.0)	30.8 (4.3)
Type‐III	34 (42.5%)	38.9 (7.4)	33.2 (5.3)	34.9 (5.8)
Type‐IV	22 (27.5%)	41.0 (7.5)	34.8 (5.8)	37.2 (5.6)
Type‐V	14 (17.5%)	41.7 (7.9)	33.9 (5.1)	35.5 (5.3)
Type‐VI	3 (3.8%)	39.6 (7.6)	33.5 (5.6)	35.4 (5.7)

Note

*P*‐values obtained with the one‐way ANOVA test: areola *P* = .133; forearm *P* = .160; sole *P* = .091

**Figure 2:**
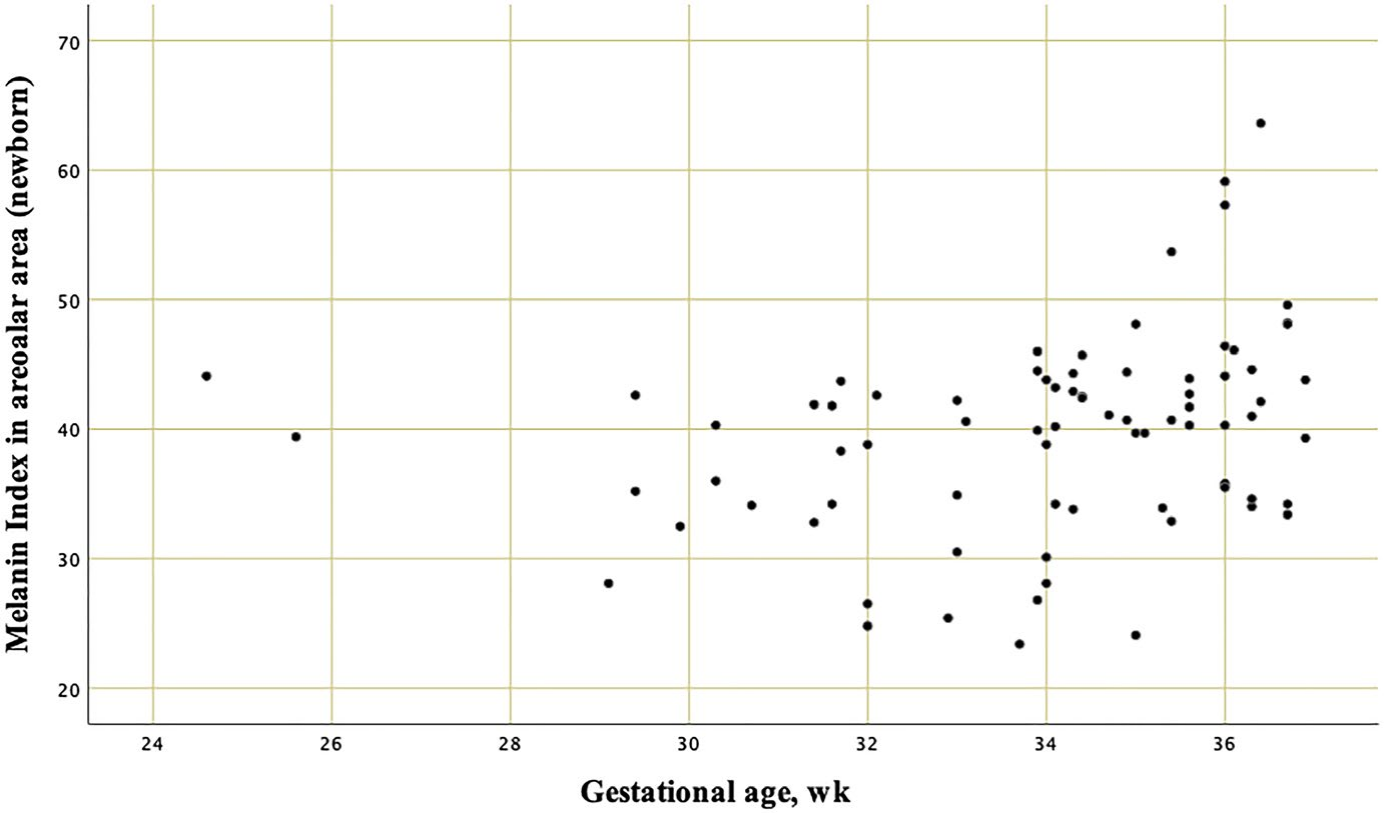
Scatter plot with values of areolar M‐Index according to gestational age calculated by early obstetric ultrasonography [Colour figure can be viewed at wileyonlinelibrary.com]

Areolar M‐Index had values plotted according to GA, Figure 2. The higher M‐Indexes were found in late‐preterm newborns. In sequence, when analyzing statistical differences, comparisons among groups of prematurity ranges revealed a significant difference in the areolar M‐Index of the group with GAs between 24 and <34 weeks, in contrast with the M‐Index of the group with ages from 34 to <37 weeks of gestation (Table 4 line 2).

**Table 4:** M‐Index of the skin of the newborn, according to gestational age and site

	n	24 to <34 wk n = 30	34 to <37 wk n = 50	*P* †
Areola, median (IQR)	78	38.3 (10.5)	41.7 (8.9)	.005
Forearm, median (IQR)	80	34.9 (10.8)	34.8 (6.7)	.773
Sole, median (IQR)	80	38.2 (9.7)	35.8 (7.3)	.227

Abbreviation: IQR, interquartile range.

^†^ Mann‐Whitney.

## 4 DISCUSSION

### 4.1 Key results and interpretation

The central contribution of this analysis was associating the color of the areola with the pregnancy chronology at birth using a quantitative assessment of melanin in the skin of neonates. The darkness of the areola, measured with an experimental photometer device, corroborated the importance of the nipple's physical modifications, already visually taken in clinical scores systems of maturity. A quantitative significant difference was present comparing newborns in the group from 24 to <34 weeks and from 34 to <37 weeks. Progressive development of the areolar skin is not new for caregivers of newborns. Before 30 weeks, the nipple is barely visible and no areola is perceptible, which markedly changes after this age.^1^ As part of maturational scores, a gradual development in the diameter of the mammalian gland, size and color of the areola are criteria used to grade maturity. A visible areola should occur simultaneously with the abundance of lanugo,^4^ smooth and fine skin 2, 4 among a set of physical and neurological parameters. However, such clinical evaluations are complex, demanding training to evaluate neurological and physical conditions to estimate GA. Though, they lacked enough accuracy and reliability in comparison with the early obstetric ultrasound dating.^15^ New post‐natal approaches are expected to combine existent parameters with new acquisitions or technology.^16^

Skin color by inspection was, in the past, recognized as an independent predictive variable of GA at birth.^21^ Regarding the skin color evaluation of newborns using optical approaches, there are scarce studies for comparisons. Previous results explored changing in the skin reflection due to pigment distribution and concentration, as well as tissue thickness according to GA.^9^ In that article, ethnicity of the infant was based on the appearance of the parents. There was a difference in the skin reflection between white and black neonates, after 32 weeks of gestation.^9^ The present study failed in demonstrating a relationship between the M‐Index assessed over the areola in premature newborns with the tonality of the skin of the mother, assessed with the Fitzpatrick scale dissimilarities. Our interpretation is that the direct measurement of the color of the skin over the areola of preterm newborns at birth may have clinical utility to support maturity evaluation, independently of ethnicity. Even the analysis did not include the fathers of the infants, a sample of mixed ethnicities in our sample allowed the evaluation of a wide range of mothers’ phenotypes of skin, varying from type‐II to type‐VI.

In other body sites such as the forearm and sole, M‐Index had no association with GA, neither with the areola pigmentation nor within variation between sites. Park & Lee (2005) found similar results, reporting no dissimilarities in the skin color of 447 newborns on the forehead, upper arm, abdomen, and inguinal area M‐indexes, neither a relationship with pregnancy dating considering the interval from 35 to 40 weeks of gestation.^10^

Physiological variations in the pigmentation of term newborns’ skin were reported according to the sex and body sites in term gestation using spectroscopy, making results difficult to compare with this sample of preterm infants 11 studied. Differently to the full term, under visual inspection, the skin of an extremely premature infant has little or no visible pigmentation, being markedly erythematous.^9^ The sample analyzed here consisted of a broad scale of prematurity from 24 weeks toward the near term, most of them inside incubators and receiving intensive neonatal care. In preterm newborns, the lack of the skin barrier is a cause of loss of water by evaporation, hypothermia, and less protection against external agents.^22^ The fragility of the premature infant's skin needs to be promptly recognized at birth to face the important clinical challenges of newborn attention. The concern of offering the best neonatal health care justifies the exploration of skin maturity based on the optical properties of this tissue.

### 4.2 Limitations and perspectives

In situ measurements of the skin pigmentation involve mathematical bio‐optical models to represent the interaction of light with the human skin. This study used the simplified principle in which the assessment of the intensity of the reflected red light is related to the optical skin density of melanin content, the M‐Index.^5^ The reasons for the three layers’ optical model assumption were previous reported to compare our results since commercial devices adopted the same model.^10^, ^23^ Despite an experimental handheld device, the repeatability of acquisitions was excellent, as previously reported.^17^ For comparability of values using the same M‐Index equation, Shriver et al^23^ found values of M‐Index varying from 30 to 76 in Europeans, East Asians, and African Americans adults, and Park & Lee reported values around 25 to 35 in Korean newborns.^10^

Another issue was the potential influence of bilirubin deposit in the skin of the newborns in the reflectance values of skin. Nonphysiologic or adaptive hyperbilirubinemia is the most common morbidity in the neonatal period, which is more severe in premature infants.^24^ In our sample, there was no phototherapy ongoing. We believe that conducting the skin examination during the first hours of life was essential to prevent possible interferences related to bilirubin accumulation in the skin. Otherwise, the external validity of this study's results is limited to the first day of the preterm infants’ life.

The present analysis brings new quantitative information comparing the relationship between neonatal maturity and M‐Index in the areola of preterm infants. Quantification of melanin provides additional information with the advantage of reliability of measurement. Variations in the colored melanin pigment are still important for the estimative correction of the presence of other skin chromophores as bilirubin.

The analysis of the M‐Index corroborated clinical antecedents of areolar darkness as a physical maturity marker in newborns. A direct quantitative value obtained by photometry is easy to acquire in birth or neonatal scenarios, just by touching the infant's skin with a small optical sensor. The timely interventions based on access to maturity of the newborn are strategic for decision‐making regarding the best neonatal care. The risk related to an immature skin barrier is essential to prevent immediate complications and future diseases during childhood.^25^ These results are not ready for a clinical proposal but highlighted the potential of the optical skin assessment to improve physical evaluation used in prematurity scores.

## 5 CONCLUSIONS

We noticed that quantification of the areolar darkness objectively evaluated the nipple's physical differences between the newborns at a cutoff of 34 weeks. The measureable M‐Index values have the potential to improve physical evaluation to assessing GA at birth.

## CONFLICT OF INTERESTS

Authors RNG and ZSNR declare a patent deposit on behalf of the Universidade Federal de Minas Gerais and Fundação de Amparo a Pesquisa de Minas Gerais, Brazil, http://www.fapemig.br/en/. The inventors were Reis, Zilma Silveira Nogueira and Guimaraes, Rodney Nascimento: BR1020170235688 (CTIT‐PN862).

## AUTHORS CONTRIBUTIONS

PCS and RGS collected clinical data, interpreted and analyzed the data, wrote and revised the article. ZSNR and RNG designed the study, interpreted and analyzed the data, wrote and revised the article.
